# Blood-Based Biomarkers in the Diagnosis of Chronic Traumatic Encephalopathy: Research to Date and Future Directions

**DOI:** 10.3390/ijms241612556

**Published:** 2023-08-08

**Authors:** Michal J. Halicki, Karen Hind, Paul L. Chazot

**Affiliations:** 1 Department of Biosciences, Durham University, Durham DH1 3LE, UK; halicki.mj@gmail.com; 2 Durham Wolfson Research Institute for Health and Wellbeing, Stockton-on-Tees TS17 6BH, UK; karenhindphd@gmail.com; 3 Department of Biosciences, Wolfson Research Institute for Health and Wellbeing, Durham University, Durham DH1 3LE, UK

**Keywords:** biomarkers, CTE, neurodegeneration, TBI, miRNA, exosomes

## Abstract

Chronic Traumatic Encephalopathy (CTE) is a neurodegenerative disease consistently associated with repetitive traumatic brain injuries (TBIs), which makes multiple professions, such as contact sports athletes and the military, especially susceptible to its onset. There are currently no approved biomarkers to diagnose CTE, thus it can only be confirmed through a post-mortem brain autopsy. Several imaging and cerebrospinal fluid biomarkers have shown promise in the diagnosis. However, blood-based biomarkers can be more easily obtained and quantified, increasing their clinical feasibility and potential for prophylactic use. This article aimed to comprehensively review the studies into potential blood-based biomarkers of CTE, discussing common themes and limitations, as well as suggesting future research directions. While the interest in blood-based biomarkers of CTE has recently increased, the research is still in its early stages. The main issue for many proposed biomarkers is their lack of selectivity for CTE. However, several molecules, such as different phosphorylated tau isoforms, were able to discern CTE from different neurodegenerative diseases. Further, the results from studies on exosomal biomarkers suggest that exosomes are a promising source of biomarkers, reflective of the internal environment of the brain. Nonetheless, more longitudinal studies combining imaging, neurobehavioral, and biochemical approaches are warranted to establish robust biomarkers for CTE.

## 1. Introduction

Neurodegenerative diseases are debilitating and are increasingly more common conditions, with dementia, their major consequence, projected to affect 139 million people worldwide in 2050 [1]. However, there is currently no cure or disease-modifying treatment for dementia. Chronic Traumatic Encephalopathy (CTE), first described in former boxers as ‘punch drunk syndrome’ [2], is an example of a condition described to cause deficits such as cognitive impairment, mental disturbance, and motor symptoms [3], which are all associated with dementia. As neurodegenerative condition management focuses on decreasing the patients’ suffering and decelerating the development of the condition [4], an early medical intervention in the case of suspected CTE could increase healthy life years by delaying the onset and worsening of these debilitating symptoms [5]. Unfortunately, the lack of clinically approved biomarkers for many neurodegenerative conditions, including CTE, translates into the inability to diagnose and intervene in the diseases’ prodromal, i.e., before the onset of symptoms, or the early stage. 

CTE has now been confirmed by post-mortem brain autopsies in many former contact sports players, such as hockey [6] and rugby [3,6,7,8,9,10], as well as deployed military personnel [3,9,11], with the history, frequency, and intensity of traumatic brain injury (TBI) being the only risk factors consistently linked with the occurrence of CTE [12]. Notably, McKee et al. [13] analyzed over 600 published cases of neuropathologically-confirmed CTE, concluding that 97% of those were associated with repetitive head impacts (RHI). However, the inability to diagnose CTE ante-mortem impedes prophylaxis, early diagnosis, and potential symptomatic treatment in the groups with a high TBI risk. This is further aggravated by non-specific initial symptoms of the disease that can be behavioral, cognitive, mood and motor-related [14,15]. Several studies reported increased incidence of poor mental health, sleep problems, cognitive impairment, and dementia in professions with high exposure to repetitive TBIs (rTBIs) [16,17,18,19], potentially indicating different stages of CTE, which emphasizes the need for biomarkers of the disease. While imaging [20] and cerebrospinal fluid (CSF) [21,22] biomarkers of CTE have been investigated with many yielding promising results, they are expensive and invasive techniques, respectively, thus limiting their use in prophylaxis and diagnosis. On the contrary, blood-based biomarkers can be easily and non-invasively collected and have already been investigated in conditions such as Alzheimer’s disease (AD) [23]. In the context of CTE, the focus of research has only recently shifted from the short-term diagnosis of TBI [24] to its long-term effects, such as CTE. While there is a growing body of studies looking at the issue, their results are often contradictory, which complicates the picture of potential blood-based biomarkers of CTE. 

So far, several reviews of TBI/CTE biomarkers have been published [25,26,27,28,29,30], but no article has focused on blood-based biomarkers of CTE, specifically. This review article aims to fill this gap by providing a comprehensive overview on the potential blood-based biomarkers of CTE, which have yielded promising results, outlining their molecular mechanisms in CTE, and emphasizing the common themes across the studies to date. Further, directions for future research will be suggested. 

## 2. Pathology of CTE and Rationale for Blood-Based Biomarkers

**Figure 1:**
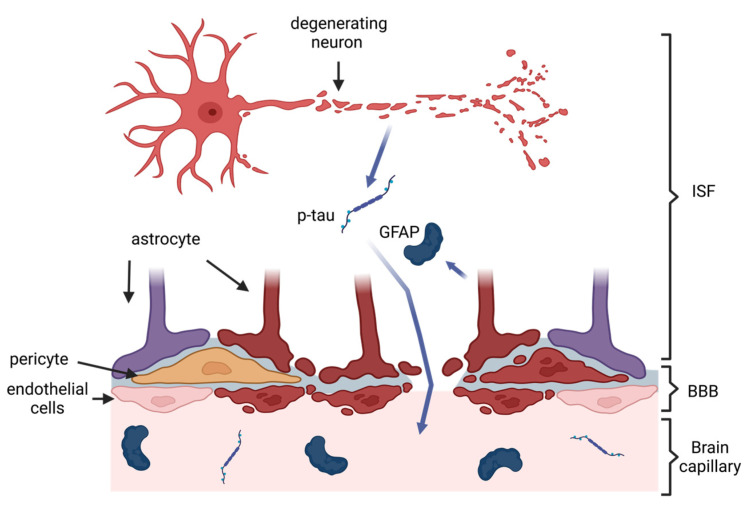
Impairment of the blood–brain barrier (BBB) following a traumatic brain injury (TBI) and a subsequent outflow of biomarkers into the systemic circulation. TBI damages axons and instigates an inflammatory response leading to efflux of neurodegenerative biomarkers, such as phosphorylated tau (p-tau) from neurons, as well as inflammation biomarkers, such as glial fibrillary acidic protein (GFAP) from astrocyte endfeet, into interstitial fluid (ISF). TBI can also damage cells maintaining the BBB (damaged cells in dark red), leading to increased permeability of the BBB and, thus, the influx of biomarkers into blood capillaries. Created with biorender.com (accessed on 20 June 2023).

CTE is a progressive tauopathy characterized by the deposition of neurofibrillary tangles (NFTs) consisting mainly of hyperphosphorylated microtubule-associated protein tau (p-tau) in the perivascular depths of cortical sulci, which increases with the progression of a disease, and is linked to trauma incurred during a TBI [31,32,33]. The aggregation of tau in NFTs induces several neurotoxic mechanisms, including microtubule destabilization, synapse loss, and potential aberrations of intracellular signaling, causing neuronal death [34]. This leads to macro-scale changes, such as brain atrophy and a consequent decrease in brain volume, in the advanced stages of the disease [35,36]. Substantial force impact on the head in a TBI causes a diffuse axonal injury (DAI), which can result in the breakage of axons and a subsequent release of axonal proteins, such as tau, into the interstitial fluid and the CSF [37,38,39,40]. On the other hand, TBI also increases the permeability of the blood–brain barrier (BBB) [41] leading to a possible efflux of axonal proteins into the systemic circulation (Figure 1). This two-way pathological mechanism allows for the detection and quantification of biomarkers from blood samples [42]. An ideal blood-based biomarker should be diagnostically accurate, i.e., be able to correctly discern between patients suffering from CTE and patients who are not; selective towards CTE, i.e., be able to discern CTE from other tauopathies, for example, AD; and feasible, i.e., be easily detectable and quantifiable from blood [43,44].

Based on the molecular pathology of CTE, two main groups of potential biomarkers can be distinguished. Potential biomarkers of neurodegeneration indicate a long-term neuronal injury and include, for instance, total (t-tau) or phosphorylated tau (p-tau). Potential biomarkers of neuroinflammation, in turn, can include glial fibrillary acidic protein (GFAP) and inflammatory cytokines, which indicate the immune activation in the central nervous system (CNS) through processes such as microgliosis or astrogliosis [27,45,46,47,48]. Further, we included a third group of biomarkers: micro RNAs (miRNA). Regardless of their role in gene regulation, all the miRNAs are discussed in a separate section due to their unique nature compared to the other reviewed biomarkers. These groups of biomarkers will be discussed in the context of: (1) the molecular basis of their role as a biomarker, (2) the studies to date, (3) the diagnostic accuracy of the biomarker, and (4) the limitations of the biomarker. 

## 3. Biomarkers of Neurodegeneration in CTE

### 3.1. Total Tau and Phosphorylated Tau 

Tau plays the most prominent role in the pathology of CTE, through the formation of NFTs and consequent neuronal dysfunction and death [34]. Numerous studies have shown a significant elevation in the extracellular p-tau deposition in the brains of people with autopsy-confirmed CTE, compared to healthy controls (e.g., Johnson et al. [49]). Most studies on the role of tau in the diagnosis of the long-term effects of TBI have focused on t-tau detectable from blood, which corresponds to compromised BBB, and p-tau, in line with signaling compromised BBB, which indicates the presence of DAI and neurotoxic mechanisms [31,32,33]. Thus far, tau has yielded promising results as an imaging biomarker [50,51], but mixed results as a biomarker from CSF [21,22,52,53]. However, both forms were shown to be significantly elevated in the brain and plasma in a mouse model of rTBIs [54]. In theory, blood tau elevation would indicate the presence of DAI, as well as its extent, which could inform diagnosis and prognosis. 

Both t-tau and p-tau concentrations analyzed from plasma have thus far yielded variable results, with many studies failing to discern between people exposed to TBIs and controls [52,55,56,57,58,59]. Nevertheless, Alosco et al. [55] reported a relationship between tau levels and RHIs, as well as plasma t-tau levels above 3.56 pg/mL, only in former rugby players despite no significance between the groups. However, others reported no relationship between t-tau and RHI [58]. Only Olivera et al. [60] observed increased plasma t-tau levels in the military deployed within the last 18 months who self-diagnosed themselves with TBIs, with a greater number of TBIs associated with a more substantial increase in plasma t-tau. As for p-tau, Vasilevskaya et al. [59] showed that tau phosphorylated at threonine 181 (p-tau181) was significantly elevated in retired contact sports athletes. Further, an abnormally high concentration of p-tau181 correlated with a decreased volume of corpus callosum (CC) and entorhinal cortex, as well as decreased fornix integrity, all of which are often observed following concussion, as indicated by imaging studies. However, Gorgoraptis et al. [52] observed no relationship between flortaucipir binding patterns in positron emission tomography (PET) scans, indicating the presence of NFTs in the brain, and plasma t-tau, which suggests that plasma t-tau is not linked to structural brain pathology. These varied results could indicate that substantial axonal damage is required for plasma tau to be significantly elevated [61]. Further, several other limitations to tau could contribute to those results, for example, a short half-life of tau in the blood [62] or the potential influence of a tau isoform from the peripheral nervous system, which current assays do not distinguish from the CNS isoform [63,64,65]. Interestingly, Gonzalez-Ortiz et al. [66] generated an antibody specifically binding the brain-derived isoform of tau, which showed high diagnostic performance in AD and was able to discriminate AD from other neurodegenerative disorders, such as frontotemporal dementia. Thus far, no studies have used this antibody in the context of rTBIs and potential CTE. 

Recently, the focus of research has extended to exosomes, which can be easily extracted from blood and overcome the issue of low blood tau concentration in the peripheral circulation and, thus, the need for ultrasensitive assays, such as Single Molecule Arrays (SiMoA) [67]. Exosomes are extracellular vesicles, which have been linked to physiological processes, such as waste excretion and cell-to-cell communication [68]. Importantly, the contents of exosomes derived from the cells in the brain are thus representing the CNS’s cellular environment, potentially serving as biomarkers that can be non-invasively quantified and are more reflective of the CTE’s underlying molecular pathology than biomarkers from the CSF. Multiple studies reported a significant difference in exosomal t-tau and p-tau [69,70,71,72,73] in people who experienced a TBI or rTBIs compared to healthy controls, with one study describing the difference in p-tau but not t-tau [74]. Moreover, several studies associated both t-tau and p-tau concentrations with post-concussive [71], neurobehavioral [70], and psychological [69] symptoms, which could indicate that exosomal tau is a symptomatic biomarker. The number of TBIs appears to be associated with both t-tau and p-tau concentrations [71], meaning that it could be reflective of the underlying molecular pathology. However, no imaging or post-mortem studies have linked the concentration of exosomal tau to structural changes in the brain so far. 

While exosomal t-tau and p-tau appear promising as blood-based biomarkers, they so far have not shown substantial specificity to CTE; a significant increase in both plasma and exosomal t-tau and p-tau can be observed, for instance, in AD [75,76,77]. Studies on tau in CTE, thus far, have focused on its diagnostic utility in discerning between people with potential CTE and healthy controls, rather than between different neurodegenerative diseases. However, Turk et al. [22] reported that tau phosphorylated at threonine 231 (p-tau231) from CSF is significantly different between CTE and AD patients, confirmed by brain autopsies. Moreover, p-tau231 was successful in distinguishing AD and CTE diagnoses. There are no studies on the long-term blood levels of p-tau231, but the protein was significantly elevated in the plasma of patients from TBI rehabilitation units with a potential chronic TBI [78]. Similarly, a post-mortem study of 473 cadavers with neuropathologically-diagnosed AD or CTE by Stathas et al. [79] showed that tau in the dorsolateral frontal cortex is differentially phosphorylated in CTE and AD; serine 202 residue (p-tau202) is significantly more phosphorylated in CTE, while serine 396 (p-tau396) residue occurs in AD, with the ratio of p-tau202 to p-tau396 being significantly higher in CTE compared to AD. Further, the number of years spent playing contact sports was a predictor of p-tau202 levels. Goetz et al. [80] reported elevated p-tau396 only in military veterans with a history of TBI and cognitive impairment (CI), and not those without CI, compared to healthy controls. Similarly, they reported increased exosomal p-tau181 in veterans with CI, regardless of their TBI status, suggesting that these epitopes cannot identify TBI-related cognitive deficits. As such, Asken et al. [76] suggest measuring plasma p-tau181 and p-tau217 to support the identification of AD pathology within Traumatic Encephalopathy Syndrome, with lower levels of these biomarkers suggesting CTE over AD. In turn, Peltz et al. [74] showed that p-tau levels were different between veterans experiencing CI with and without a history of TBIs, yet the exact epitope measured was not mentioned in the publication. These studies suggest only a couple of differentially expressed p-tau epitopes, among more than 30 tau phosphorylation sites [81]. Investigation into the feasibility of p-tau202, p-tau231, and other under-researched epitopes of p-tau as a blood-based biomarker of CTE should be continued to assess their diagnostic performance and specificity to CTE.

### 3.2. Amyloid Beta

While Amyloid beta (Aβ) plaques are primarily associated with AD, TBI has been shown to increase the concentration of the Amyloid Precursor Protein and Aβ peptides in the brain tissue and CSF. These proteins can foster the formation of plaques [9,82,83], which are toxic to brain cells and trigger neurodegenerative processes [84]. The presence of Aβ plaques in cadavers with neuropathologically-diagnosed CTE has been reported, but it is not as universal as the deposition of NFTs in CTE and has been linked to the possession of the Apoϵ4 allele, as well as significantly older age at death, potentially indicating Aβ plaques’ association with old age in CTE [9].

Thus far, the results for the blood-based Aβ peptide have been mixed. From plasma, Lebjman et al. [85] reported a significant increase in Aβ40 and a trend for increased Aβ42 in military personnel who experienced TBI, deployed a minimum of 16 months before the investigation, while other studies reported no significant changes in different groups of athletes who experienced TBIs [56,58]. Exosomal Aβ peptides, in turn, appear more promising, with a significant increase of Aβ42 in groups that experienced TBIs [70,72], with one study showing no difference [74]. While more research is needed to confirm the role of exosomal Aβ as a biomarker, its specificity to CTE is greatly limited. Goetz et al. [80] showed that Aβ42 was elevated in war veterans with CI regardless of whether they experienced TBI in the past, yet the significance was greater for veterans with both CI and TBI. Unexpectedly, however, Turk et al. [22] showed that CSF Aβ42 was lower in people with confirmed CTE than in healthy controls. Moreover, the difference in CSF Aβ42 was able to distinguish between patients with CTE and AD. These results suggest that CSF Aβ42 levels could show selectivity towards CTE, as well as contradict the studies on exosomal Aβ42. More research is required to elucidate this relationship. 

### 3.3. Neurofilament Light

Neurofilaments are intermediate filaments expressed exclusively in neurons. While their exact function remains to be elucidated, they are thought to play a critical role in axonal stability. Therefore, the efflux of neurofilaments into the CSF and potentially systemic circulation is indicative of neuroaxonal injury and has been suggested as a biomarker of neurological disorders, such as Parkinson’s disease (PD) or AD [86]. In the context of TBI, the research has focused on the diagnosis of TBI through plasma NfL, reporting a significant elevation in plasma NfL following a TBI that predicted clinical outcomes [86,87,88,89,90]. However, knowledge about the long-term relationship between NfL and CTE is scarce. In a rat model, a single blast overpressure exposure was not shown to significantly increase plasma NfL 10 months after a blast simulation, but there was a trend for increased NfL in exposed rats compared to controls [91]. Two studies showed no difference in plasma NfL between athletes who experienced rTBIs and those who did not [56,59], with another study reporting no increase in exosomal NfL one-year post-TBI [92]. On the contrary, Peltz et al. [74] showed that exosomal NfL was significantly elevated in veterans diagnosed with CI, both in patients with and without a history of TBIs compared to healthy controls, while there was no difference in exosomal NfL for veterans with a history of TBI but no CI in relation to controls. Importantly, exosomal NfL was significantly elevated in veterans with CI and a history of TBIs compared to veterans without CI but with a history of TBIs, which suggests that NfL could be a symptomatic biomarker. Further, Dickstein et al. [91] observed that while there was no difference in plasma NfL concentration when veterans were compared to a control group, the highest levels of NfL were in veterans with excess [18F]AV1451 PET ligand retention, which reflected the anatomical distribution of tauopathy in CTE observed in post-mortem studies. Also, Shahim et al. [57] reported that serum NfL remained elevated for up to five years after a single TBI, with the protein correlating with brain structural and neuronal damage measured by diffusion tensor imaging and magnetic resonance imaging (MRI). Similarly, Vasilevskaya et al. [59], despite no significant difference in the plasma concentration, showed an inverse relationship between plasma NfL levels and hippocampal and CC volume, as well as the white matter integrity of fornix. These results indicate that blood-based NfL could be representative of structural brain pathology caused by TBI.

The major limitation of NfL is its lack of specificity to CTE. Asken et al. [93], analyzing a group of nine cadavers, showed that elevated NfL could be observed in patients with different neuropathologically confirmed neurological disorders, such as Frontotemporal Lobar Degeneration, CTE and AD. Nonetheless, further research into the chronic effects of TBIs and their relationship to NfL is warranted to establish the clinical relevance and selectivity of NfL in the context of CTE. 

### 3.4. Other Biomarkers of Neurodegeneration 

There have been individual studies investigating several other potential biomarkers of neurodegeneration. Ubiquitin C-Terminal Hydrolase L1 (UCH-L1) is an abundant protein in the brain and is essential to the proper maintenance of axonal integrity. Its dysfunction has been implicated in neurodegeneration, where it can, for example, misfold and constitute NFTs in AD [94]. As such, it was found to be significantly elevated in the CSF of AD patients [95,96]. In potential CTE, CSF UCH-L1 was associated with grey matter abnormalities in long-term TBI survivors, but there was no difference between this group and controls [52]. However, no UCH-L1 elevation, as well as no correlation with brain structural changes, was reported in patients with TBIs compared to controls, both from plasma [57] and exosomes [72], thus far. Similarly, alpha-synuclein, which aggregates into Lewy bodies in disorders, such as PD, has been shown to cause Lewy Body Disease concomitant to CTE in the brains of deceased contact sports athletes [97,98]. However, only one study has looked at alpha-synuclein so far, showing no difference in exosomal alpha-synuclein concentration between veterans with and without a history of TBIs [74]. Also, Goetz et al. [72,80] noted a significant increase in the exosomal cellular prion protein (PrPc), synaptogyrin-3, and aquaporin-4. In addition, they show that the levels of PrPc and synaptogyrin-3 proteins were only increased in CI veterans, both with and without a history of TBI, yet not in a group with a history of TBI but no CI. However, no further studies on these proteins were conducted.

## 4. Biomarkers of Neuroinflammation in CTE

While the microglia activation and peripheral immune cell recruitment following a TBI has been shown, evidence is now emerging that chronic neuroinflammation might have an intrinsic role in the pathogenesis and progression of CTE through the induction of secondary neuronal injury [99,100,101,102,103]. A post-mortem study by Johnson et al. [45] showed that reactive microglia could be detected up to 18 years following a single TBI, which also coincided with white matter degeneration. Similarly, an in vivo MRI study reported increased neuroinflammation in several brain regions with hippocampal atrophy in retired rugby players [104]. In a mice model, Loane et al. [105] observed elevated microglial activation in the cortex, CC, and thalamus for up to 1 year following a moderate blast injury associated with neurodegeneration and increased biomarkers of neuroinflammation. These initial reports suggest that biomarkers of neuroinflammation could be indicative of CTE, as well as its progression, but the findings are not conclusive yet and require further research. 

### 4.1. Glial Fibrillary Acidic Protein 

GFAP is an intermediate filament protein and a major cytoskeletal component of astrocytes, which maintain synaptic transmission and axonal metabolism [106]. Following TBI, astrocytes mediate processes, such as BBB permeability and the inflammatory response [107]. Astrocyte immune activation is accompanied by an increase in the expression of GFAP [108]. On the other hand, an astrocytic injury could cause the efflux of GFAP [109]. Therefore, GFAP could represent both chronic neuroinflammation and neurodegeneration in CTE. Its blood elevation has been shown to relate to structural abnormalities in imaging studies after mild TBI [110,111,112]. Shahim et al. [57] reported that serum GFAP was significantly increased in chronic TBI patients for up to 5 years after a single TBI compared to controls, but showed little association with structural brain change. Other studies showed no difference in the plasma levels of GFAP between professional athletes with a history of RHI and post-concussive syndrome [58], retired athletes with a history of TBIs [56], and their respective controls. However, exosomal GFAP yielded more promising results, with Flynn et al. [92] reporting a significant increase in a group of patients one-year post-TBI, and Peltz et al. [74] showing that veterans with a history of TBIs and CI had elevated exosomal GFAP, while veterans with a history of TBIs but without CI did not. This suggests that GFAP can be representative of functional changes and symptoms. Nonetheless, only a single imaging and no post-mortem studies have analyzed GFAP levels in relation to structural brain abnormalities. Yet, a small pilot post-mortem study of nine patients with different neuropathological diagnoses showed that especially high GFAP was present in the brains with AD neuropathologic changes. This requires further exploration, in larger cohorts [93]. 

### 4.2. Inflammatory Cytokines 

The increased activation of microglia, which has been shown to occur following TBIs, upregulates the production of several pro-inflammatory cytokines. These lead to increased permeability of the BBB, elevated secretion of chemokines that cause the migration of peripheral leukocytes into the brain, as well as the production of reactive oxygen species, which altogether foster neuroinflammation and can trigger secondary cell death. Inflammatory cytokines investigated in the context of the long-term consequences of TBI involve IL-6, IL-10, and TNF-α, which can all be secreted by microglia, indicating microgliosis [99]. As these can be expressed in all the tissues, the concentration of the cytokines was quantified from neuron-enriched exosomes. Peltz et al. [74] analyzed all three molecules and showed that IL-6 was significantly elevated both in veterans with CI but no history of TBIs, as well as in those with a history of TBIs compared to healthy controls. Moreover, in the concussed veterans, the IL-6 and TNF-α levels were significantly higher in those experiencing CI. In turn, the IL-10 levels were not different between the groups. Goetzl et al. [72,80] also showed significantly increased IL-6 in high-impact sports students with at least two mild TBIs, as well as military veterans with a history of TBIs and symptoms of CI. Surprisingly, Gill et al. [70] reported no difference in either IL-6 or TNF-α between military personnel who experienced a TBI within the past 3 years and controls, while noting that IL-10 was significantly elevated in the former group. However, no study so far has analyzed the relationship between the levels of these cytokines and structural changes in imaging and post-mortem studies. 

## 5. Micro RNA Biomarkers in CTE

Micro RNAs (miRNAs) are small non-coding RNAs, which regulate a variety of processes at the post-transcriptional level. The expression of miRNAs can change in response to different physiological and pathological states [113]. Specifically, several studies identified panels of miRNA biomarkers from saliva [114] and blood [68,115,116,117] that showed different levels of specific miRNAs between patients following a TBI and controls. As such, they showed great potential in diagnosing TBI. A great advantage of miRNAs over conventional protein panels is their stability; Gilad et al. [113] showed that their levels did not change after four hours at room temperature, while two freeze–thaw cycles affected their levels to a small extent. Further, the target miRNAs can be easily quantified using conventional Real-Time Quantitative Reverse Transcription PCR (qRT-PCR), which overcomes the requirement for ultrasensitive assays, like SiMoA [67], or exosome extraction [68], to reliably quantify proteins, such as tau. 

However, only a handful of studies have looked at the expression of miRNAs in potential CTE patients, thus far. Alvia et al. [118] compared the expression of different miRNAs previously indicated in the prefrontal cortex of brains donated by people who suffered from either CTE, Amyotrophic Lateral Sclerosis (ALS), or both. While much of the expression of miRNAs overlapped between CTE and ALS, they identified several miRNAs specific to CTE, which were involved in cell growth, apoptotic and inflammatory pathways. As per biological fluids, Ghai et al. [119] used next-generation sequencing (NGS) to compare the miRNA profiles of plasma and extracellular vesicles (EV) between veterans with a history of chronic TBI and controls. They detected significant differences in the levels of multiple previously described, as well as novel, miRNAs, which they confirmed using qRT-PCR. They also observed that most miRNAs were circulating freely in plasma, which supports the use of plasma without the need for EVs isolation. Ge et al. [120] compared serum and exosome biomarkers between 12 patients with a history of rTBIs and, thus, a different likelihood of CTE and respective controls, distinguishing serum and exosomal miR-1183 and exosomal miR-297 as potential diagnostic miRNA biomarkers of CTE. Neither of these studies related miRNA expression to neurobehavioral or imaging evidence. Further, due to few studies, miRNAs especially promising for CTE diagnosis cannot be distinguished yet. Considering the potential advantages of miRNAs over conventional protein biomarkers, further research is warranted. 

## 6. Discussion and Future Directions

Although the number of studies on the potential biomarkers of CTE has been increasing in recent years, the research is still in its early stages, with common themes, such as little selectivity towards CTE diagnosis, as well as conflicting results, now being reported. The studies so far differ in the cohorts employed and the underlying cause of the concussions. Veterans primarily suffer from blast-related brain injuries, while contact sports athletes suffer from sports-related brain injuries, which have been shown to have a distinct pathological mechanism. While both the TBI types cause secondary nerve injury [121], the distinct primary mechanism could lead to different concentrations of biomarkers. Further, studies greatly vary in the duration from the last TBI, from some studies having participants one-year post-TBI [58], to others on average 37 years post-TBI [74]. Similarly, the average number of TBIs is variable, with some researchers looking at the long-term effects of up to 3 TBIs [92] and others having participants with a history of 10 TBIs, on average [56]. Also, some studies were based on self-reported accounts of TBI, or occupancies and sport positions with high susceptibility to TBI, which were not supported by medical documentation (e.g., [59,69,74,85]). However, in contrast to a recent statement by the Concussion in Sport Group [122], there is evidence to demonstrate the effect of head impacts on the biomarker levels described in this review, indicative of a causal link between TBI and CTE and long-term neurological effects [13,28,59,69,70,71,72,73,74,123,124]. Specifically, Nowinski et al. [124] used the Bradford Hill criteria to analyze the causality between RHI and CTE. Based on several factors, including the consistent relationship between RHI and CTE in analyses from multiple brain banks, while CTE is rarely confirmed without RHI, and the absence of alternative hypotheses with plausible evidence, a causative link between RHI and CTE was concluded. Further, there is an overlap between patterns of p-tau histochemical staining in post-mortem brain slices of CTE patients and topographic tau accumulation in PET scans of people with suspected CTE [28]. Also, this pathological hallmark molecule of CTE is consistently increased in the blood [59] and blood exosomes [69,70,71,72,73,74] of individuals with a history of TBIs compared to controls. In fact, a recently published analysis, which concluded that 97% of 600 confirmed cases of CTE were associated with repetitive head impacts, only strengthens this argument [13].

Moreover, some experimental protocols differed between the described studies, especially concerning the methods of extraction and purification of the exosomes. Several methods of extraction were used, ranging from size exclusion chromatography and ultracentrifugation to using different extraction kits, which all have been shown to result in variable yield and purity [125]. Further, different collection procedures and handling of samples, such as the time between blood draw and centrifugation, the speed, and the size of the needle, all affect exosome yield and purity [68]. Moreover, not all the studies enriched their yield for neuron-derived exosomes (e.g., Kenney et al. [71]; Muraoka et al. [73]). Research into the differences in biomarker concentrations from neuron-enriched and crude systemic exosomes is warranted to establish the most optimal way for exosome isolation for the study of CNS biomarkers. Similarly, while most studies analyzed plasma biomarkers, some measured their concentration from serum, which contains lower levels of, for instance, tau than plasma [57]. Notably, most research has been carried out in predominantly male cohorts, which prevents the generalization of the results to the whole population, as some studies suggested sex differences in neurobehavioral outcomes and biomarker levels following TBI [126]. 

**Figure 2:**
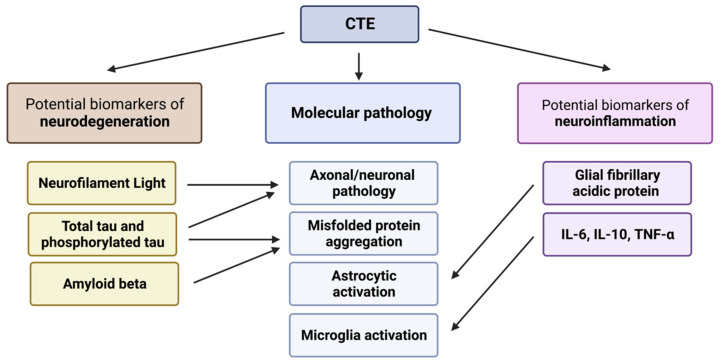
Summary of the potential protein biomarkers that showed promising results in their use for CTE diagnosis, with the molecular pathology they indicate. Created with biorender.com (accessed on 20 June 2023).

Considering the evidence, several protein biomarkers show promise for the diagnosis of CTE (Figure 2). Also, despite limited evidence so far, miRNAs appear promising and should be investigated further. Yet, the general problem with potential CTE biomarkers is their role in multiple diseases and, thus, the lack of specificity to CTE. So far, p-tau molecules phosphorylated on epitopes seemingly specific to CTE, such as p-tau202, and, potentially, certain miRNAs, appear to show the greatest promise in diagnosing CTE, specifically. Further, biomarkers specific to AD or other neurodegenerative diseases could aid differential diagnosis. Research into a panel of neurodegenerative biomarkers specific to various diseases with overlapping symptoms is warranted, too. For instance, Peltz et al. [74] showed that a panel of exosomal p-tau, NfL, GFAP, IL-6, and TNF-α was able to distinguish between CI veterans with and without a history of TBIs, as well as veterans with a history of TBIs and symptoms of CI and those without these symptoms. Generally, exosomes appear to be an especially promising new tool, as they are useful for detecting biomarkers that are expressed systemically and, thus, are not CNS-specific, for instance, inflammatory cytokines. Further, exosomes readily cross the BBB [68], hence there is no need for substantial BBB damage to detect biomarkers from blood. The greatest limitation in most of the discussed studies, both in plasma/serum- and exosome-based biomarkers, is their cross-sectional nature, with only some including structural analysis using imaging techniques, and very few confirming suspected diagnoses by post-mortem investigation. Since the disparity in results can be caused by the heterogeneity of long-term effects of TBI, which cause not only CTE, but also PD, AD, ALS, or a combination of these diseases [31,36], there is a need for longitudinal and multidimensional cohort studies combining neurobehavioral, biochemical, imaging, as well as neuropathological approaches. Recently, two such studies have begun [127,128]. This way the levels of biomarkers can be associated with structural and functional brain pathology, establishing robust biomarkers of CTE, aiding diagnosis of the disease, and potential preventative and symptomatic treatment. However, a substantial amount of time will pass until the results and conclusions from these longitudinal studies are published. Meanwhile, further cross-sectional studies in cohorts at risk of developing CTE, especially investigating the exosomal concentrations of different biomarkers and associating them with structural abnormality in imaging studies and functional abnormality in neurobehavioral studies, are warranted.
